# Air Pollution Is Associated with COVID-19 Incidence and Mortality in Vienna, Austria

**DOI:** 10.3390/ijerph17249275

**Published:** 2020-12-11

**Authors:** Hans-Peter Hutter, Michael Poteser, Hanns Moshammer, Kathrin Lemmerer, Monika Mayer, Lisbeth Weitensfelder, Peter Wallner, Michael Kundi

**Affiliations:** 1 Center for Public Health, Department of Environmental Health, Medical University of Vienna, 1090 Vienna, Austria; hans-peter.hutter@meduniwien.ac.at (H.-P.H.); hanns.moshammer@meduniwien.ac.at (H.M.); kathrin.lemmerer@meduniwien.ac.at (K.L.); lisbeth.weitensfelder@meduniwien.ac.at (L.W.); peter.wallner@meduniwien.ac.at (P.W.); michael.kundi@meduniwien.ac.at (M.K.); 2 Department of Hygiene, Medical University of Karakalpakstan, Nukus 230100, Uzbekistan; 3 Institute of Meteorology and Climatology, University of Natural Resources and Life Sciences Vienna, 1180 Vienna, Austria; monika.mayer@boku.ac.at

**Keywords:** COVID-19, PM10, NO_2_, spatial air pollution differences, incidence, mortality

## Abstract

We determined the impact of air pollution on COVID-19-related mortality and reported-case incidence, analyzing the correlation of infection case numbers and outcomes with previous-year air pollution data from the populations of 23 Viennese districts. Time at risk started in a district when the first COVID-19 case was diagnosed. High exposure levels were defined as living in a district with an average (year 2019) concentration of nitrogen dioxide (NO_2_) and/or particulate matter (PM10) higher than the upper quartile (30 and 20 µg/m^3^, respectively) of all districts. The total population of the individual districts was followed until diagnosis of or death from COVID-19, or until 21 April 2020, whichever came first. Cox proportional hazard regression was performed after controlling for percentage of population aged 65 and more, percentage of foreigners and of persons with a university degree, unemployment rate, and population density. PM10 and NO_2_ were significantly and positively associated with the risk of a COVID-19 diagnosis (hazard ratio (HR) = 1.44 and 1.16, respectively). NO_2_ was also significantly associated with death from COVID-19 (HR = 1.72). Even within a single city, higher levels of air pollution are associated with an adverse impact on COVID-19 risk.

## 1. Introduction

Air pollution has been linked to an increased risk of respiratory infections (see reviews by Ciencewicki and Jaspers [1], Domingo and Rovira [2]). With regard to the epidemic of SARS-CoV in 2003, an ecological study from China reported case fatality rates to be higher in regions with higher air pollution, as measured by the Air Pollution Index (API) [3]. However, in another study the outbreak of SARS-CoV in mainland China was found to be not related to API [4]. Kan et al. [5] found daily SARS-CoV-2 mortality associated especially with nitrogen dioxide (NO_2_).

Based on these initial findings, a possible causal association between air pollution and COVID-19 mortality has been debated. Conticini et al. [6] argued that the high level of air pollution in Northern Italy could be a cofactor for the higher COVID-19 prevalence and mortality in this region by weakening the upper airways’ defense and leading to chronic inflammation. Ogen [7] investigated COVID-19 fatality rates in 66 administrative regions in Italy, Spain, France, and Germany in relationship to long-term NO_2_ concentrations. Most of the fatalities were found in the regions with the highest NO_2_ concentrations. Another ecological study [8] reported an association between long-term exposure to particulate matter (PM_2.5_) and COVID-19 incidence in Canada. Highly significant correlations between PM_2.5_ concentrations and COVID-19 incidence, mortality, and case fatality rates were also shown for the Italian provinces [9]. Moreover, the role of PM in the spread of COVID-19 was emphasized in a review focusing on Italian cities [10]. A systematic review of 15 studies concluded that especially PM_2.5_ and NO_2_ are contributing to COVID-19 spread and lethality [11].

Villeneuve and Goldberg [12] recently analyzed the methodology of epidemiological studies investigating the association between air pollution, and SARS and COVID-19 coronavirus outbreaks. They pointed out uncertainties because of possible exposure measurement error and residual confounding.

While a single study is always prone to error, multiple studies undertaken in different settings and at different scales allow for a more holistic appraisal of the environmental impact of air pollution on infection risk. Due to these considerations, we set out to investigate whether in a large city such as Vienna, with a known variance of air pollution across districts, an association with local incidence and mortality of COVID-19 can be found. Vienna has 23 districts that range from green spaces close to Vienna Forest to areas with industry and heavy traffic. We obtained daily numbers of cases and deaths for each of these districts and district-wide air pollution data for the year 2019 as indicators of chronic exposure. Daily incidence and mortality were analyzed by Cox regression considering district data of population attributes as confounders.

## 2. Materials and Methods

### 2.1. Epidemiological and Demographic Data Sources

The analysis was based on daily reported COVID-19 cases and deaths collected at the epidemiological documentation system of the Vienna Health Authority until 21 April (after peak of infections), 2020. The first diagnosis of COVID-19 in Vienna was reported on 28 February 2020. A first fatality was attributed to COVID-19 in Vienna on 11 March 2020. New infections were reported with the date of first diagnosis or death, and the district of residence. The city of Vienna, with a total population of 1,893,779 as of 31 October 2018, consists of 23 districts. Population numbers for each district were extracted from Statistics Austria general population data sets [13], and population density was calculated using municipal district data. Sociodemographic data per district retrieved included: percentage of persons aged 65 or above, percentage of foreigners, unemployment rate, and percentage of persons with a university degree.

### 2.2. Air Pollution Data Sources

The European Environment Agency (EEA) collects and provides data sets of specific pollutant concentrations of all EU member states. Nitrogen dioxide (NO_2_) and particulate matter (PM_10_ and PM_2.5_, particulate matter which passes through a size-selective inlet with a 50% efficiency cutoff at 10 μm or 2.5 µm aerodynamic diameter, respectively) data sets for all Viennese sites for the year 2019 were retrieved from the EEA’s database on 13 March 2020. From these data sets, the annual average levels of NO_2_ and PM_10_ were calculated, based on daily mean values from sites with at least 75% data availability. Although the city of Vienna operates a dense air quality monitoring network consisting of 17 sites [14], observations are not carried out in each of the 23 urban districts. Thus, we estimated district level NO_2_ and PM_10_ annual means via a representative-site approach, based on spatial proximity of districts to observational sites and results from previous Differential Optical Absorption Spectroscopy (DOAS) measurements of NO_2_ in Vienna [15]. Schreier et al. 2019 [15] investigated the correlation between the car DOAS zenith sky measurements and the in situ observations from the air pollution monitoring network in Vienna. They found both measurements to be highly correlated (R = 0.4). Retrospective data for years earlier than 2019 revealed declining trends, but districts with high exposures in 2019 also displayed high exposures in the previous years. Ranking between districts was less constant among the districts with lower average NO_2_ concentration. Therefore, we examined the effect of living in a district with high pollution (binary exposure variable). Because representative measurements could not be obtained for PM_2.5_ for all districts, analyses were based on NO_2_ and PM_10_ only.

### 2.3. Statistical Analysis

During the observation period, Austria entered a period of restricted social interactions (lockdown). Most shops and all restaurants and schools were closed, and people were encouraged to work in home offices and abstain from traveling. The introduction of SARS-CoV-2 into a district was considered a random event. Residents of that district were assumed to be at risk of acquiring COVID-19 or of dying with a diagnosis of COVID-19 beginning with the date when the virus was introduced into the district. Thus, a Cox proportional hazard model was built based on all residents of each district at risk, beginning at the date of the first diagnosed case in that district, and they were followed until diagnosis or death, or until the end of the observation period (21 April 2020), whichever came first.

Average air pollution concentration (NO_2_, PM_10_) in those districts in 2019 was assumed to be the independent factor of interest; population density, percentage of persons aged 65 or above, percentage of foreigners, unemployment rate, and percentage of persons with a university degree were included in the model as possible confounders. As for some districts, NO_2_ and PM_10_ concentrations could only be estimated, air pollution concentration was entered as a binary variable equal or above versus below the upper quartile of all districts (20 µg/m^3^ for PM10, 30 µg/m^3^ for NO_2_).

## 3. Results

**Figure 1:**
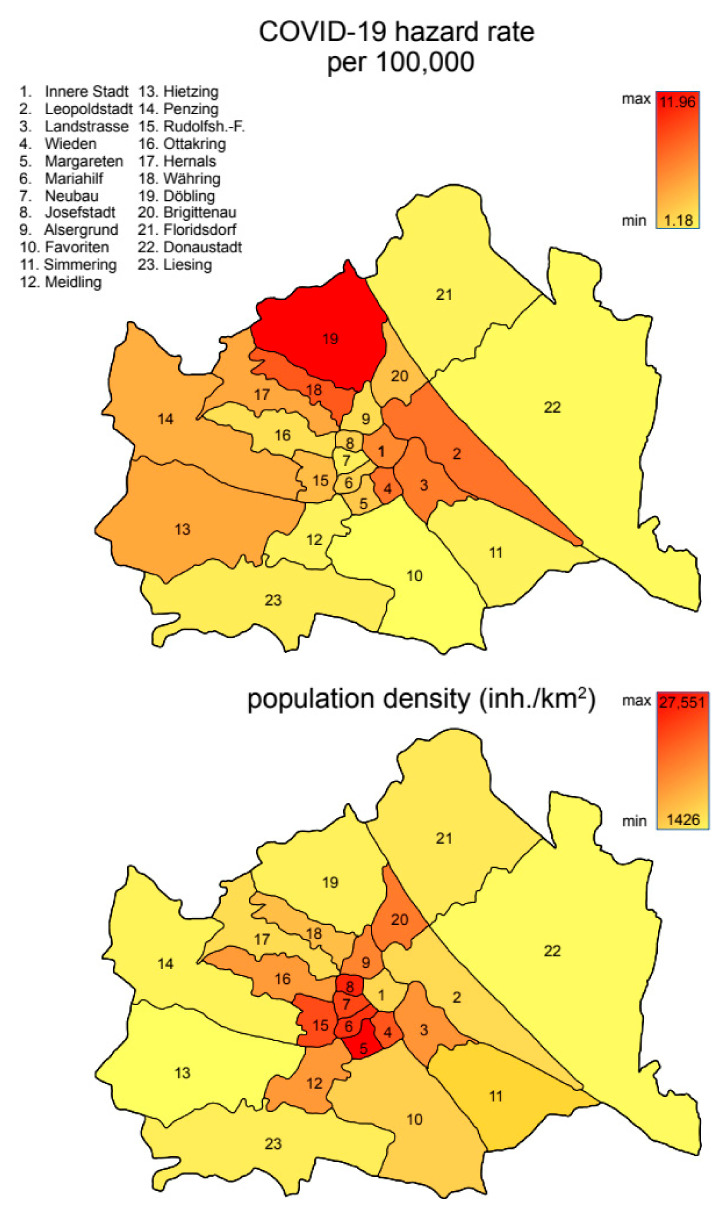
Map of Vienna districts with color-coded indication of COVID-19 hazard rate per 100,000 inhabitants (**top**) and population-density inhabitants per km^2^ (**bottom**).

The first COVID-19 case was diagnosed on February 28 in the 22nd district. The last district to report a case was the 6th district on March 19. In the observation period, case numbers as well as the local hazard rate (considering the time of first case in district) were found to be different in districts of Vienna (Figure 1). COVID-19 deaths did not occur in all districts within the observation period. Overall, 1665 cases and 59 deaths were reported during the observation period, the first on March 21 in the 2nd district.

Additional area level factors were chosen a priori, driven by availability and existing evidence from the literature [16,17]. Indeed, some factors showed some correlation with air pollution levels, rendering them possible confounders (Table 1).

**Figure 2:**
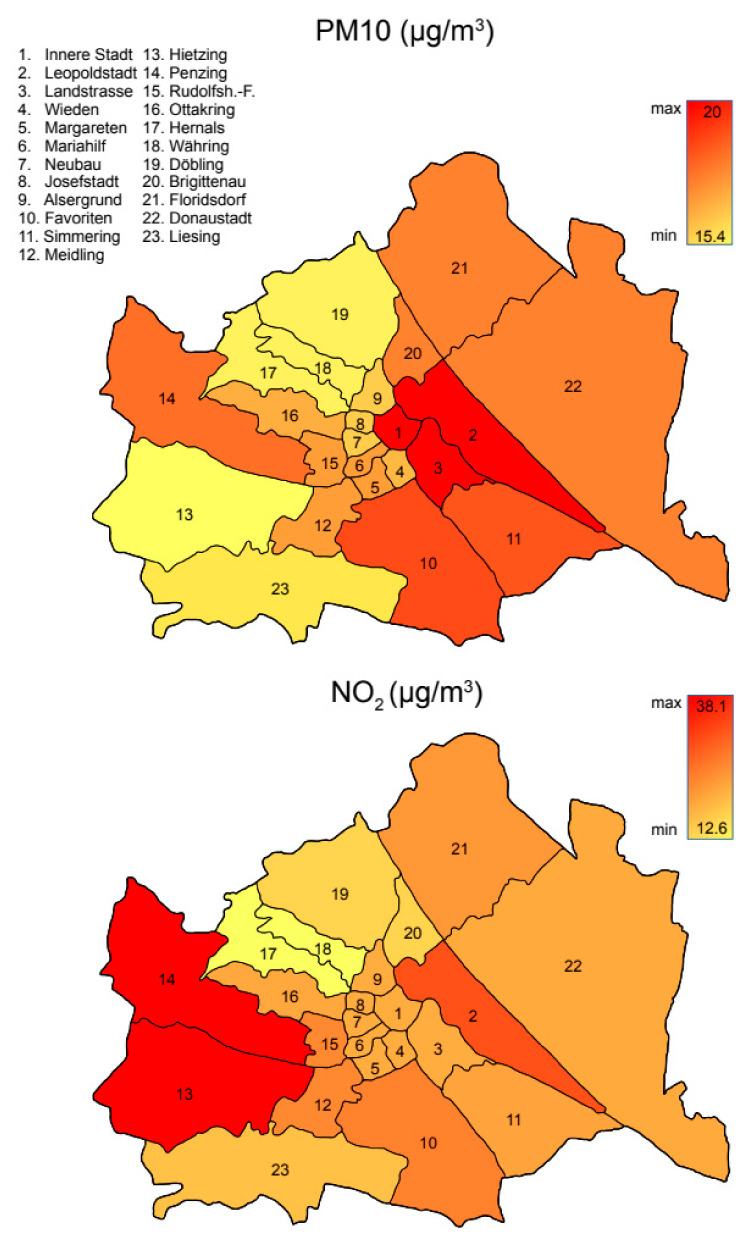
Map of Vienna districts with color-coded indication of mean annual airborne particulate matter particles smaller than 10 µm, PM10, (**top**), and NO_2_, (**bottom**) in µg/m^3^.

Air pollution levels measured as 2019 annual mean values of PM10 and NO_2_ did differ between districts (Table 2, Figure 2), allowing for a meaningful analysis. In the Cox regression, both air pollutants increased the risk for being diagnosed with COVID-19 (hazard ratio (HR) being 1.44 and 1.16 for the upper quartile of PM10 and NO_2_, respectively) and for dying with that diagnosis (HR = 1.72); however, for COVID-19 deaths, only NO_2_ was statistically significant (Table 3).

Population density appeared to be a protective factor, while a higher percentage of persons with university degrees and unemployment rates (the latter only significant for cases, not deaths) increased the risk.

## 4. Discussion

The city of Vienna maintains a dense air quality monitoring network that has been used to examine health impacts of air pollution before [18]. The density of pollution-monitoring stations allows for an analysis of air pollution effects on COVID-19 infections and deaths on the basis of rather small urban areas. Data analysis was facilitated by reduced individual movements of inhabitants among districts during the individual (“stay-at-home” order) and economic lockdown of Austria between 16 March 2020, and the middle of May [19], as schools and most shops were closed, office workers were encouraged to work in home offices, and public transport systems were limited.

We found increased risks of being diagnosed with COVID-19 and of dying from that disease with increasing concentrations of air pollutants (PM10, NO_2_) in the home district averaged over the year 2019. An association between air pollution and risk of respiratory infections has been reported before [20,21,22] and does have some biological plausibility. Indeed, both short-term and long-term impacts of air pollution on risk and severity of SARS-CoV-2 infection are conceivable. For a short-term association, several possible mechanisms have been proposed. There could be a direct interaction between air pollutants and the virus in the atmosphere. Virus attached to particulates could be protected from UV radiation degradation [23], ozone [24], or other environmental factors, thus prolonging the viability of the virus in the environment. Particulates could serve as carriers of the virus, enhancing the deposition of the virus in the lung [25]. Particulates and irritant gases could affect the mucous membranes of the respiratory tract, rendering the cells more vulnerable to infection. While these mechanisms would mostly act around the time of infection, and also during the incubation period and early stages of the disease, damage to the mucous membranes could lead to a more severe disease outcome. Chronic exposure to air pollution is a known risk factor for respiratory diseases [26,27,28] such as emphysema and bronchial obstruction through remodeling of lung tissues. These chronic respiratory diseases could render a person more susceptible to infection and certainly are risk factors for a more severe course of the disease in case of an infection.

Studies also support the hypothesis that air pollution induces overexpression of ACE-2 [9,10,29]. Both air pollution and COVID-19 can cause systemic disease effects, and therefore, a combined effect also on mortality risk is plausible.

Similar findings were reported in other studies. Acute responses were analyzed by Zhu et al. [30]. They found (data of 120 cities) that a 10 μg/m^3^ increase (lag0–14) in PM2.5, PM10, NO_2_, and O_3_ was associated with a 2.24% (95% CI: 1.02 to 3.46), 1.76% (95% CI: 0.89 to 2.63), 6.94% (95% CI: 2.38 to 11.51), and 4.76% (95% CI: 1.99 to 7.52) increase in the daily counts of confirmed cases, respectively.

US data were analyzed by Wu et al. [31]. COVID-19 death counts for more than 3000 counties in the United States were related to long-term average PM2.5 adjusted for 20 potential confounding factors. An increase of 1 μg/m^3^ in PM_2.5_ was associated with an 8% increase in the COVID-19 death rate. It was concluded that a “small increase in long-term exposure to PM2.5 leads to a large increase in the COVID-19 death rate”.

While an air-pollution-dependent increase in mortality related to an infection of the lung may be expected, the correlation with case incidence may appear less obvious. However, a link between infection vulnerability and air quality has already been postulated for the 1952 London smog; numerous reports have confirmed these findings since, while the exact mechanisms are still unknown [32].

Pansini and Fornacca [33] showed higher rates of SARS-CoV-2 infections in areas with high PM2.5, NO_2_, and CO levels (China, Italy, US). The relationship between poor air quality and COVID-19 incidence and mortality was strongest in Italy. The analyses of Fattorini and Regoli [34] revealed that long-term exposure to major pollutants (NO_2_, O_3_, PM_2.5_, PM_10_) significantly correlated with cases of COVID-19 in up to 71 Italian provinces. They underlined that besides current concentrations, the chronicity of exposure may influence the exceptional variability of SARS-CoV-2 infection frequency in Italy.

The given epidemiological setting does not allow for a clear separation between effects of acute and of chronic exposure. A person living in a district with higher air pollution concentration in 2019 will, on average, likely also more often experience higher exposure levels on any single day in 2020. Examining the temporal course of effects in a time-series analysis would in principle allow for disentangling the contribution of acute and chronic exposures. However, this would require a substantial variation in daily concentrations over the period of study. This was not the case for the rather short period of analysis in our case. Furthermore, the temporal course of the number of daily cases was heavily influenced by measures on the individual and on the whole society’s level to curb the epidemic. The lockdown led to a reduction in air pollution [35,36]; therefore, we focused on chronic effects and the air quality data of the preceding year. We are pretty confident that the year 2019 was representative, in that areas with higher air pollution levels in 2019 had higher levels also in the previous years. Absolute values changed between years, with generally a declining trend for both pollutants in the last 10 years or longer. However, the ranking of districts remained fairly stable. Examining longer time periods would also only lead to spurious improvements in exposure assessment. We might know better the longer-term concentration in each district. However, for our COVID patients, we only know their recent address. The farther back the pollution data, the less certain their place of living is.

As we aimed to test the hypothesis that chronic exposure leads to changes in the airways that render airways more susceptible to viral infection, we think that average exposure, expressed as annual mean levels, is the best descriptor of chronic exposure. Examining multiple sets of parameters would only lead to the statistical problem of multiple testing.

A model making use of spatial variation in exposure seems more viable and has also been applied by previous studies on air pollution and COVID-19 risk. These previous studies examined case numbers per population numbers, while our study looked at case numbers per person time. Nevertheless, in a separate paper, we have attempted to also investigate the temporal association between air pollution and COVID-19 cases in Vienna (submitted elsewhere).

Researchers have long been struggling with the fact that in experimental studies in humans or animals, health effects started to be observed at rather high exposure levels, while in epidemiological research, clear associations were already visible at much lower levels. Different explanations have been brought forward for that apparent discrepancy [37]: Experimental human or animal studies usually apply small sample sizes. Therefore, these studies might lack the necessary power to detect subtle effects. In the experimental setting, often a very homogeneous group is exposed. In real-life settings, exposed people display a large variability in individual susceptibility that is not captured by the experimental setting. However, even if susceptible groups were investigated, effects were typically seen at much higher levels. More likely, the discrepancy between experimental and epidemiological findings is due to the experiments typically investigating a single pollutant, while under real-world conditions, people are exposed consecutively or simultaneously to multiple factors. An infectious agent in combination with air pollutants might serve as a good model of such a combined exposure.

We initially expected a more pronounced spread of the virus in the more densely populated districts. To our surprise, the opposite was the case. However, a recent study from 913 US metropolitan counties also observed a protective effect of population density, at least for COVID-19 mortality [38]. It remains to be observed whether people living in denser areas maybe pay more attention to self-protection measures such as keeping minimum distance or wearing masks. In the case of Vienna, an additional cause might also lay within the fact that densely inhabited areas (districts 6–8, 15) are located within the inner city, consisting of mostly residential areas, whereas in outer districts, industry and wasteland prevail, lowering overall density. However, population density still seems to remain a factor worth investigating, also representing an indicator for socioeconomic status. Hence, a study focusing on population density alongside other socioeconomic indicators is in preparation.

In addition, the observation of both districts with a higher percentage of persons with unemployment (for COVID-19 incidence) and with a university degree (for both incidence and mortality) deserves further comments. As Villeneuve and Goldberg [12] have pointed out, communities with a better socioeconomic status might have better access to health care, ensuring a higher testing rate. This would lead to a spurious increase in case numbers in richer districts, but not necessarily to an increase in mortality. We propose that especially in the initial phase of an epidemic, population segments with higher income, which are also often better-connected and more mobile, could have an increased risk of infection. In the later stages of an epidemic, crowding and poor access to health care might tip the balance. We concentrated on the early stage of the epidemic on purpose because this included the first lockdown where mobility between districts also was reduced to a minimum. However, mobility could still have differed between social strata. COVID-19 cases and mortality in districts might also be influenced by the presence of large retirement homes and of clusters in the homes.

Unfortunately, the database does not allow for stratified analyses and the inclusion of additional potential confounders. Thus, we could not control for possible individual confounding factors but only included area-level factors in our analysis. However, we are confident that this analysis adds additional input to the ongoing discussion on air pollution and infectious diseases.

## 5. Conclusions

Even in a single large city with local variation in air pollution we found an association between chronic exposure to increased levels of NO_2_ and PM_10_, and COVID-19 incidence and mortality. The daily hazard was about 40% higher both for incidence and for death from COVID-19 if PM_10_ was above 20 µg/m^3^. NO_2_ was an independent risk factor for both incidence and mortality, and especially was found to elevate the hazard for daily deaths by more than 70%. Some evidence has already indicated that SARS-CoV-2 infection and mortality are influenced by air pollution, maybe due to the chronic airway impairment it induces. That such a relationship can also be found in a city with a rather limited exposure gradient and one that has experienced low numbers of infection is surprising and points to a greater impact of air pollution on respiratory infection than previously assumed.

## Figures and Tables

**Table 1:** Cross-correlation between predictors (upper triangle Pearson correlation, lower triangle Spearman correlation). Correlations with a coefficient of determination >10% highlighted in bold.

	PM_10_ > 20 µg/m^3^	NO_2_ > 30 µg/m^3^	Percent Aged 65+	Percent Foreigners	Unemployment Rate	Percent University Degree	Population Density (per km^2^)
PM_10_ > 20 µg/m^3^		0.243	−0.072	0.214	0.164	−0.155	−0.050
NO_2_ > 30 µg/m^3^	**0.422**		0.160	−0.132	0.056	−0.127	−0.216
Percent aged 65+	−0.090	−0.101		−**0.629**	−**0.512**	**0.323**	−**0.543**
Percent foreigners	0.216	0.018	−**0.630**		**0.526**	−0.097	**0.613**
Unemployment rate	0.308	**0.364**	−0.307	**0.541**		−**0.861**	−0.042
Percent university degree	−0.226	−**0.386**	0.121	−0.085	−**0.847**		**0.365**
Population density (per km^2^)	0.041	−0.255	−**0.618**	**0.674**	−0.037	**0.383**	

**Table 2:** Descriptive statistics of the 23 Viennese districts.

District	Population Number (N)	Size (km^2^)	Density (N/km^2^)	Cases (N)	Hazard Rate per 100,000	Deaths (N)
1	16,306	2.86	5683	24	6.21	0
2	104,946	19.23	5457	132	7.10	6
3	91,745	7.39	12,402	80	6.72	3
4	33,263	1.77	18,741	31	7.10	1
5	55,407	2.01	27,551	31	3.52	0
6	31,864	1.45	21,939	19	3.11	0
7	32,288	1.60	20,121	22	1.93	0
8	25,466	1.08	23,368	21	3.15	0
9	41,958	2.96	14,142	39	2.69	1
10	204,142	31.81	6416	133	1.18	4
11	103,008	23.25	4430	71	1.66	3
12	97,634	8.10	12,051	75	3.20	4
13	53,778	37.70	1426	57	4.95	0
14	92,990	33.75	2755	84	4.73	6
15	77,621	3.92	19,786	73	4.04	0
16	103,785	8.67	11,967	102	2.54	4
17	57,292	11.38	5031	54	4.90	0
18	51,587	6.34	8129	44	8.23	1
19	72,947	24.94	2925	136	11.96	8
20	86,502	5.70	15,153	69	3.80	2
21	165,673	44.43	3728	146	1.67	8
22	191,008	102.28	1867	147	1.38	6
23	106,281	32.06	3315	75	2.01	2
Sum	1,897,491	414.79		1665		59
Mean	82,499.60	18.03	10799	72.39	4.25	2.56

**Table 3:** Effect estimates from Cox regression model.

	SARS-CoV-2 Positive			COVID-19 Death		
	HR	95% CI		HR	95% CI	
PM_10_ > 20 µg/m^3^	**1.44**	**1.25**	**1.65**	1.49	0.73	3.08
NO_2_ > 30 µg/m^3^	**1.16**	**1.05**	**1.29**	**1.72**	**1.02**	**2.90**
Percent aged 65+	1.03	1.00	1.06	0.93	0.78	1.10
Percent foreigners	1.01	0.98	1.03	0.92	0.80	1.06
Unemployment rate	**1.10**	**1.03**	**1.18**	1.46	0.97	2.22
Percent university degree	**1.05**	**1.03**	**1.07**	**1.14**	**1.01**	**1.28**
Population density (per km^2^)	**0.98**	**0.96**	**0.99**	**0.91**	**0.84**	**0.99**

HR: Hazard ratio; CI: Confidence interval; bold indicates statistical significance (*p* < 0.05).
